# Supplementary material for: Thermal Ablation Compared to Partial Hepatectomy for Recurrent Colorectal Liver Metastases: An Amsterdam Colorectal Liver Met Registry (AmCORE) Based Study
Source: Cancers (Basel). 2021 Jun 2;13(11):2769. doi: 10.3390/cancers13112769 (PMC8199651; doi:10.3390/cancers13112769)
Supplement: Supplementary file 1 [file cancers-13-02769-s001.zip › cancers-1224565-supplementary.pdf]

**Table S1.** Univariable and multivariable cox regression analysis to detect potential confounders associated with local tumor progression-free survival (LTPFS). After removal of initial CRLM diagnosis and number of recurrent metastasis, and adjusting for the confounder time between initial treatment and diagnosis recurrence, corrected HR of repeat local treatment was 1.486 (95% CI, 0.594–3.714; $p = 0.397$).

| | | **Univariable Analysis** | | **Multivariable Analysis** | |
|---|---|---|---|---|---|
| | | **HR (CI)** | ***p*-Value** | **HR (CI)** | ***p*-Value** |
| Repeat local treatment | Repeat resection | Reference | 0.959 | Reference | 0.397 |
| | Repeat thermal ablation | 1.023 (0.426–2.454) | | 1.486 (0.594–3.714) | |
| **Patient-Related Factors** | | | | | |
| Gender | Male | Reference | 0.655 | | |
| | Female | 1.233 (0.492–3.089) | | | |
| Age (years) | | 1.006 (0.970–1.044) | 0.730 | | |
| ASA physical status | 1 | Reference | 0.263 | | |
| | 2 | 1.713 (0.230–12.747) | | | |
| | 3 | 0.645 (0.067–6.208) | | | |
| Comorbidities | None | Reference | 0.776 | | |
| | Minimal | 1.288 (0.568–2.919) | | | |
| | Major | 0.857 (0.191–3.838) | | | |
| BMI (kg/cm$^2$) | | 1.065 (0.979–1.159) | 0.141 | | |
| Primary tumor location | Rectum | Reference | 0.715 | | |
| | Colon left-sided | 0.678 (0.268–1.720) | | | |
| | Colon right-sided | 0.819 (0.297–2.262) | | | |
| **Factors Regarding Initial Local Treatment of CRLM** | | | | | |
| Initial CRLM diagnosis | Synchronous | Reference | **0.002** | Reference | 0.077 |
| | Metachronous | 3.964 (1.643–9.560) | | 2.391 (0.909–6.284) | |
| Number of tumors | 1 | Reference | 0.898 | | |
| | 2–5 | 1.273 (0.454–3.574) | | | |
| | >5 | 1.150 (0.365–3.625) | | | |
| Size of largest metastasis (mm) | Small (1–30) | Reference | 0.353 | | |
| | Intermediate (31–50) | 0.399 (0.114–1.390) | | | |
| | Large (>50) | * | | | |
| Extrahepatic disease | No | Reference | 0.545 | | |
| | Yes | 0.538 (0.072–4.004) | | | |
| Type of procedure | Resection | Reference | 0.950 | | |
| | Thermal ablation | 1.018 (0.401–2.585) | | | |
| | Resection and thermal ablation | 0.693 (0.264–1.820) | | | |
| | IRE | * | | | |
| | SBRT | * | | | |
| **Factors Regarding Repeat Local Treatment of CRLM** | | | | | |
| Time between initial treatment and diagnosis recurrence (months) | | 1.029 (1.010–1.049) | **0.003** | 1.032 (1.012–1.052) | **0.001** |
| Number of tumors | 1 | Reference | **0.016** | Reference | 0.194 |
| | 2–5 | 0.292 (0.126–0.677) | | 0.431 (0.173–1.072) | |
| | >5 | * | | * | |
| Size of metastasis (mm) | Small (1–30) | Reference | 0.525 | | |
| | Intermediate (31–50) | 1.914 (0.624–5.871) | | | |
| | Large (>50) | * | | | |
| Chemotherapy | No | Reference | 0.264 | | |
| | Yes | 0.592 (0.236–1.485) | | | |
| Margin size | <5mm | Reference | 0.652 | | |
| | >5mm | 0.717 (0.169–3.042) | | | |

HR = hazard ratio, CI = 95% confidence interval, ASA = American Society of Anesthesiologists score, BMI = body mass index, * = insufficient subgroup size for each treatment group.

**Table S2.** Univariable and multivariable cox regression analysis to detect potential confounders associated with distant progression-free survival (DPFS). After removal of age and initial CRLM diagnosis, and adjusting for the confounders size of largest recurrent metastasis and time between initial treatment and diagnosis recurrence, corrected HR of repeat local treatment was 1.024 (95% CI, 0.545–1.922; $p$ = 0.942).

| | | Univariable Analysis | | Multivariable Analysis | |
|---|---|---|---|---|---|
| | | **HR (CI)** | ***p*-Value** | **HR (CI)** | ***p*-Value** |
| Repeat local treatment | Repeat resection | Reference | 0.660 | Reference | 0.942 |
| | Repeat thermal ablation | 1.139 (0.637–2.038) | | 1.024 (0.545–1.922) | |
| **Patient-Related Factors** | | | | | |
| Gender | Male | Reference | 0.834 | | |
| | Female | 1.052 (0.655–1.691) | | | |
| Age (years) | | 0.982 (0.962–1.003) | **0.092** | 0.986 (0.964–1.009) | 0.239 |
| ASA physical status | 1 | Reference | 0.470 | | |
| | 2 | 1.425 (0.570–3.562) | | | |
| | 3 | 1.748 (0.664–4.607) | | | |
| Comorbidities | None | Reference | 0.649 | | |
| | Minimal | 1.015 (0.648–1.590) | | | |
| | Major | 1.344 (0.707–2.554) | | | |
| BMI (kg/cm$^2$) | | 0.971 (0.919–1.027) | 0.305 | | |
| Primary tumor location | Rectum | Reference | 0.877 | | |
| | Colon left-sided | 1.008 (0.606–1.677) | | | |
| | Colon right-sided | 1.134 (0.643–2.000) | | | |
| **Factors Regarding Initial Local Treatment of CRLM** | | | | | |
| Initial CRLM diagnosis | Synchronous | Reference | **0.089** | Reference | 0.199 |
| | Metachronous | 0.691 (0.452–1.058) | | 0.731 (0.453–1.179) | |
| Number of tumors | 1 | Reference | 0.311 | | |
| | 2–5 | 1.077 (0.645–1.798) | | | |
| | >5 | 1.504 (0.843–2.684) | | | |
| Size of largest metastasis (mm) | Small (1–30) | Reference | 0.189 | | |
| | Intermediate (31–50) | 0.630 (0.379–1.046) | | | |
| | Large (>50) | 0.731 (0.291–1.840) | | | |
| Extrahepatic disease | No | Reference | 0.238 | | |
| | Yes | 0.577 (0.232–1.437) | | | |
| Type of procedure | Resection | Reference | 0.824 | | |
| | Thermal ablation | 1.203 (0.717–2.017) | | | |
| | Resection and thermal ablation | 0.889 (0.529–1.496) | | | |
| | IRE | 1.108 (0.263–4.668) | | | |
| | SBRT | 0.698 (0.095–5.153) | | | |
| **Factors Regarding Repeat Local Treatment of CRLM** | | | | | |
| Time between initial treatment and diagnosis recurrence (months) | | 0.980 (0.963–0.998) | **0.032** | 0.973 (0.951–0.995) | **0.016** |
| Number of tumors | 1 | Reference | 0.235 | | |
| | 2–5 | 1.422 (0.934–2.164) | | | |
| | >5 | 1.852 (0.254–13.511) | | | |
| Size of largest metastasis (mm) | Small (1–30) | Reference | **0.008** | Reference | **0.002** |
| | Intermediate (31–50) | 1.533 (0.839–2.803) | | 1.959 (1.054–3.641) | |
| | Large (>50) | 8.469 (1.982–36.183) | | 10.409 (2.266–47.816) | |
| Chemotherapy | No | Reference | 0.158 | | |
| | Yes | 0.707 (0.437–1.144) | | | |

HR = hazard ratio, CI = 95% confidence interval, ASA = American Society of Anesthesiologists score, BMI = body mass index.
